# Comparative Analysis of Drugs Frequently Suspected of Causing Adverse Drug Reactions Reported via the Spontaneous Reporting System Versus in a Prospective Multicentre Cohort Study in Hospital Emergency Departments

**DOI:** 10.3390/jcm14175921

**Published:** 2025-08-22

**Authors:** Verena Graeff, Markus Wehler, Harald Dormann, Julia C. Stingl, Katja S. Just, Albrecht Eisert

**Affiliations:** 1 Department of Interprofessional Hospital Development, University Hospital Augsburg, 86156 Augsburg, Germany; verena.graeff@uk-augsburg.de; 2 Central Emergency Department, University Hospital Augsburg, 86156 Augsburg, Germany; markus.wehler@uk-augsburg.de; 3 Central Emergency Department, Hospital Fürth, 90766 Fürth, Germany; harald.dormann@klinikum-fuerth.de; 4 Ordinary Member of the Drug Commission of the German Medical Association (AkdÄ), 10623 Berlin, Germany; 5 Department of Clinical Pharmacology and Pharmacoepidemiology, Heidelberg University Hospital, 69120 Heidelberg, Germany; juliacarolin.stingl@med.uni-heidelberg.de; 6 Institute of Clinical Pharmacology, RWTH Aachen University Hospital, 52062 Aachen, Germany; kjust@ukaachen.de

**Keywords:** spontaneous reports, drug safety, adverse drug reaction, prospective cohort study, emergency department

## Abstract

**Background/Objectives**: Pharmacovigilance aims to identify, assess, and minimize drug risks, with spontaneous reporting playing a central role. However, the high level of underreporting and the varying data quality are limitations that should be minimized through prospective cohort studies. **Methods**: Spontaneous reports reported to the Drug Commission of the German Medical Association (AkdÄ) over one year were compared with the adverse drug reaction (ADR) cases systematically recorded in hospital emergency departments. The frequencies of the demographic patient characteristics and the odds ratios, as the relationship between suspected and concomitant medication, were calculated. **Results**: In the spontaneous reports, cases were reported by patients a median of 12 years younger, and the group of older patients was less represented (10.8% versus 27.3% in the prospective cohort study). Within the study, cases with polypharmacy were documented significantly more often (a median of seven drugs [IQR 3;10] versus a median of two drugs [IQR 1;5] in the spontaneous reports). New drugs and drugs discussed in the media were frequently reported as causing ADRs, whereas drugs with an effect on the central nervous system were more often suspected in the emergency department setting. **Conclusions**: Both sources for ADRs provide complementary information that improves the detection of risk signals. The aim for the future is to further increase the awareness of spontaneous reports and to answer specific questions with the help of structured investigations. It is important to compare and validate the findings of spontaneous reports and investigations in regular intervals to improve drug safety.

## 1. Introduction

Pharmacovigilance aims to identify, understand and prevent adverse drug reactions (ADRs) [1]. This can be achieved through marketing authorization studies, direct monitoring [2] or spontaneous reports [3]. Healthcare professionals (regardless of their specialization or experience) are obliged to report suspected cases in accordance with their professional code of conduct [4,5]. However, a survey has shown that only 43% of the general practitioners were aware of this obligation [6]. Nevertheless, these reports accounts for the majority of all spontaneous reports [7]. Patients are also encouraged to report suspected cases. The proportion and significance of these reports has not been definitively clarified [7], but it appears that patients describe ADRs in more detail and as having a greater impact on their daily lives [8].

In Germany, reports can be made either to the drug commission of the respective professional association (medical profession/pharmacists) or directly to the federal authority (BfArM for Drugs and Medical devices and PEI for Blood products and Vaccines). Companies with marketing authorization are also obliged to maintain a pharmacovigilance system and conduct evaluations. All suspected cases reported by all parties within the European Union are collected in the EudraVigilance database of the European Medicine Agency (EMA) and analyzed for risk signals [9].

Spontaneous reports are considered as a conceptually simple, inexpensive and universally applicable tool [2,7]. Risk signals can be quickly detected on the basis of a small number of reports, avoiding potential dangers at an early stage [7]. Therefore, spontaneous reports are considered to be the cornerstone of the pharmacovigilance system [3,7,10]. Their main limitations are the varying data quality and the low reporting rate (‘underreporting’) [3,6,11]. Suspected ADRs are reported via a digital questionnaire, where only a few fields, such as date of birth, gender, ADR and suspected drug, are mandatory, and all other fields, such as concomitant drugs, examination results (e.g., laboratory values) and medical history, are optional [12] (Figure S1). This may explain the high variance in the completeness of reports. A study showed that 12.7% of all reports were classified as “well-documented” and 18.8% as “poorly documented”, with a statistical correlation between high-quality recording and the severity of the event [3]. In addition, it has been scientifically proven that underreporting accounts for up to 95% of all suspected cases [11], and one survey showed that only 16.6% of all general practitioners reported an ADR within one year [6]. It should be noted that spontaneous reports can be recorded in various ways; therefore, reports to the AkdÄ represent only a fraction of all spontaneous reports. This must be considered as a further limitation. Another point to consider is the causality assessment. Spontaneous reports are often suspected cases that are not validated at the time of reporting. The subsequent evaluation of the temporal context and the accumulation of similar reports results in a risk signal, making it possible to detect new, previously unknown ADRs [12].

This data gap can be closed by prospective case–cohort studies [2] or retrospective surveillance data [10]. The requirements for prospective data collection are high and thus this process is costly, time-consuming and requires trained personnel [2]. However, the high data quality and good detection rate [2] make it possible to quantitatively answer specific scientific questions.

The aim of this descriptive analysis was to compare two different methods for recording ADRs—spontaneous reports and prospective cohort studies—in terms of their similarities and differences (e.g., demographic data, suspected drug groups) with the aim of assessing the feasibility of detecting risk signals. The authors are not aware of any comparable scientific work. The limitations of both methods must be continuously evaluated to further develop pharmacovigilance systems, adapt to changing conditions such as big data, and to increase drug safety.

## 2. Materials and Methods

### 2.1. Comparison Groups

All cases reported to the Drug Commission of the German Medical Association (AkdÄ) in 2017, excluding reports on blood products and vaccines, were used for the spontaneous reports. This year is in the middle of the data collection period for the multicentre prospective cohort study ADRED (Adverse Drug Reactions in Emergency Departments), which took place between 2015 and 2018 (Phase I).

The prospective multicentre cohort study ADRED systematically collected ADRs in emergency departments at four university and tertiary care hospitals in Germany (Hospital Fürth, University Hospital Ulm, University Hospital Bonn and the Robert Bosch Hospital Stuttgart). The study was based on a study plan and was conducted by specially trained healthcare professionals (physicians and pharmacists) between 2015 and 2018 (Phase I). Data from adult patients (18 years or older) who presented to the emergency department due to an ADR were collected. These cases were assessed by emergency staff and/or study personnel. Whether the patient was hospitalized or discharged after emergency treatment was irrelevant, and patients who intentionally harmed themselves or cases of medication error were excluded. Written informed consent was obligatory for all patients, except for those who were unable to give consent due to the severity of their ADR (e.g., intubated, comatose). The study personnel documented extensive demographic data, the severity of ADR, suspected and concomitant drugs and diagnoses on an electronic Case Report Form (eCRF). The high number of mandatory fields ensured a high documentation rate regardless of the study centre. Training and regular telephone conferences ensured a defined causality assessment and a high level of consistency over all study centres. In Figure S2, the recruitment flowchart is shown. The method and results of the ADRED study (Phase I) have already been published in detail [13,14,15].

### 2.2. Data Adjustment

**Figure 1:**
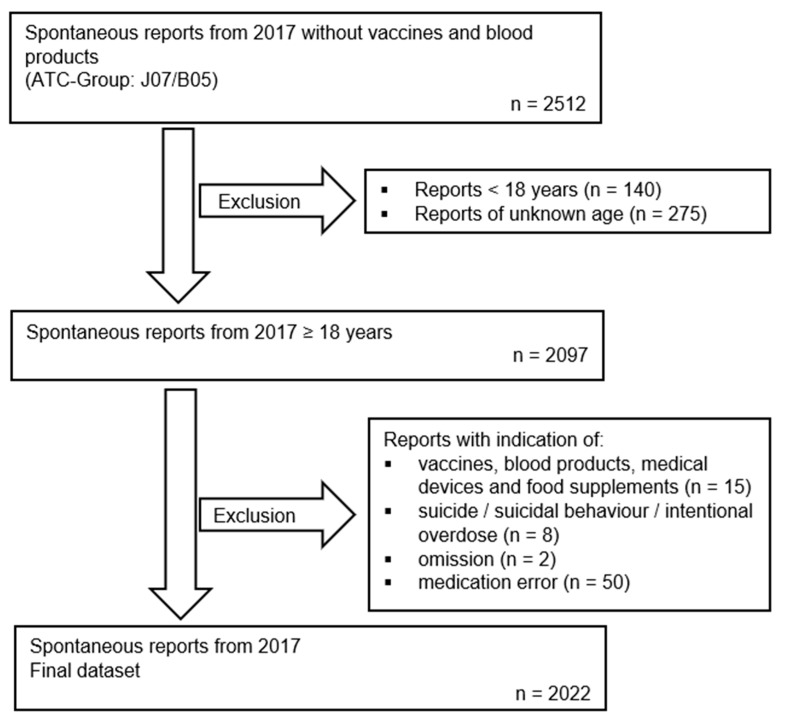
Flowchart of data adjustment for spontaneous reports from 2017, including the inclusion and exclusion criteria.

To ensure comparability between the two groups, the data were adjusted, and spontaneous reports were classified manually by the AkdÄ, analogous to the ADRED study data (Phase I). This led to the exclusion of minors, reports of unknown age, reports of intentional self-harm (suicide/suicidal behaviour and intentional overdose), omission cases, i.e., cases in which patients did not adhere to treatment and cases with medication errors such as prescription errors, and reports with dispensing and/or administration errors. Reports that were falsely recorded based on vaccines or blood products were also excluded, as were cases involving medical devices and dietary supplements without detailed information on ingredients. Cases with the “suicidal ideation” ADR were not excluded if there was no active self-harming behaviour. The process of data adjustment is shown in the flow chart in Figure 1.

### 2.3. Data Classification

The severity of ADRs is classified as non-serious or serious. Serious reactions were subdivided into death, life-threatening, hospitalization, disability and other serious reactions (only in the spontaneous reports). Combination drugs were divided into their individual active ingredients to calculate the number of these ingredients. The drugs were classified using the anatomical–therapeutic–chemical (ATC) classification [16].

### 2.4. Statistical Analysis

For the comparison, demographic and clinical data such as age, sex, number of suspected and taken drugs and the severity of the ADR were descriptively analyzed. Participants were divided into three age groups: adults (18–64 years), young-old (65–79 years) and old-old (≥80 years). Categorical variables are given as absolute and percentage values, while continuous variables are given as medians and interquartile ranges [IQRs] due to a lack of a normal distribution in the Kolmogorov–Smirnov test.

To estimate the probability of a drug being reported or recorded as suspected or concomitant in the respective population (spontaneous reports or ADRED study), the corresponding odds ratios (ORs) with 95% confidence interval (95% CI) were calculated. The 95% CI was corrected using Bonferroni. An OR with a 95% CI greater than 1 indicates that the drug group was disproportionately frequently reported or recorded as suspected. When the value is 1, the ratio is balanced and when the value is less than 1, the drug group is found disproportionately frequently in the concomitant medication. Statistical significance was set at α ≤ 0.05. The statistical software used was SPSS^®^, version 26–28, from IBM.

## 3. Results

The comparative analysis was based on 2022 reports from the AkdÄ and 2215 cases from the ADRED study (Phase I). In the ADRED study, patients were on average 12 years older (median 61 vs. 73 years) and took a median of 7 [IQR 3;10] drugs per day [14] versus 2 [IQR 1;5] drugs for patients in the spontaneous reporting system. In the spontaneous reports, the number of reported drugs increased significantly with age (Spearman rank correlation: 0.260, *p* < 0.001). In most cases in both spontaneous reports (median 1 [IQR 1;1]) and emergency department admissions (median 1 [IQR 1;2]), one drug was suspected to have caused the ADR [14]. An overview of the main data for both groups can be found in Table 1.

**Figure 2:**
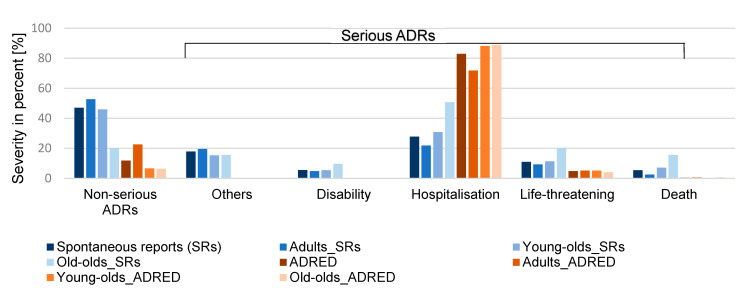
Distribution of severity within the spontaneous reports and the ADRED study—total and for the three age groups. ADRs: adverse drug reactions.

Almost 50% of all spontaneous reports were classified as non-serious versus 12% in the ADRED study [14]. The reason for this can be found in the written consent of the patient required for the study [14]. In the spontaneous reports, there is an increasing proportion of serious events (except for “other serious events”) with increasing age. For example, the proportion of deaths increased sixfold between adults and the old-olds (2.5% versus 15.5%). This was not observed in the ADRED study, where the proportion of serious events is more balanced [15]. The distribution of the age groups in relation to the severity is shown in Table 1 and is graphically illustrated in Figure 2.

**Figure 3:**
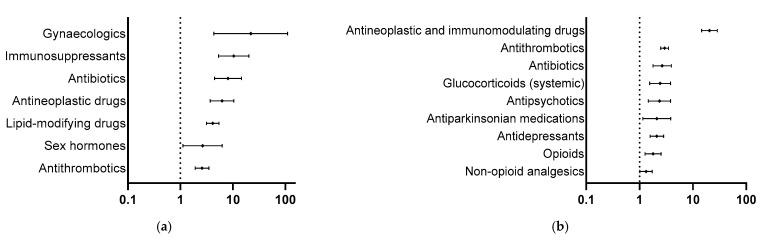
Drug groups with an OR (95% CI) > 1 for (**a**) spontaneous reports and (**b**) the ADRED study.

The most reported frequently taken drugs in the spontaneous reports were lipid-modifying drugs, antithrombotics, ACE inhibitors/AT-1 antagonists, antidiabetics and beta blockers (12.1%, 8.4%, 8.0%, 7.0%, and 5.7%, respectively). For the ADRED study, these were antithrombotics, diuretics, beta blockers, ACE inhibitors/AT-1 antagonists and drugs for acid-related diseases (10.4%, 7.6%, 6.7%, 6.6% and 6.6%, respectively) [14]. In this respect, the drug groups that are most often suspected of causing an ADR are lipid-modifying drugs, antithrombotics, antineoplastic drugs, antibiotics and immunosuppressants (21.2%, 13.0%, 7.9%, 7.4% and 7.2%, respectively) in the spontaneous reports and antithrombotics, antineoplastic and immunomodulating drugs, beta blockers, diuretics and non-opioid analgesics (19.1%, 14.8%, 7.2%, 7.1% and 5.2%, respectively) for the ADRED study [14]. The drug groups with a high probability of having a corresponding OR (95% CI) of >1 are summarized in Table 2 and graphically illustrated as forest plots in Figure 3a,b [14].

There are similarities between antineoplastic drugs, antibiotics and antithrombotics. Differences can be seen for gynecological drugs and sex hormones, lipid-modifying drugs and drugs affecting the nervous system. In the spontaneous reports, intrauterine contraception with progestogen (levonorgestrel) represents the majority of gynecological drugs, observed in 53 of 56 reported cases. Hormonal contraceptives for systemic use, consisting of a fixed combination of progestogen and estrogen with dienogest/ethinylestradiol (*n* = 10) and levonorgestrel/ethinylestradiol (*n* = 6), were reported as sex hormones. The statins atorvastatin (*n* = 107), simvastatin (*n* = 65) and fluvastatin (*n* = 43) were suspected of having triggered an ADR in almost half of all cases (44.5%), followed by ezetimibe (*n* = 54) and the PCSK-9 inhibitors evolucumab (*n* = 46) and alirocumab (*n* = 43). In the ADRED study, the five most frequently suspected drugs as having an influence on the nervous system are citalopram (*n* = 42), oxycodone (*n* = 37), metamizole (*n* = 34), pregabalin (*n* = 32) and mirtazapine (*n* = 28) [14].

## 4. Discussion

For both methods, it was shown that drug groups with a narrow therapeutic range (= range between the minimal therapeutic efficacy and the minimal toxicity), such as antineoplastic, immunomodulating, antibiotic and antithrombotic drugs, are frequently suspected of causing ADRs [14]. Cytostatic drugs, due to their physiological effects, are well known as a drug class with a high rate of severe ADRs [17,18]. The same applies to antithrombotics. The physiological effect of antithrombotics on the tendency to bleed is therapeutically desirable but, at the same time, is a risk factor for ADRs [19,20]. The pronounced awareness and the good detectability may explain the high detection rate in both groups.

Spontaneous reports often included drugs that were either new to the market or that had been discussed in the media. For example, drugs with an influence on lipid metabolism accounted for a high percentage. New drug groups, such as PCSK-9 inhibitors, are approved through the benefit assessment procedure within the Joint Federal Committee (G-BA) in accordance with §35a SGB V. Dietary and lipid-lowering therapeutics, particularly statin drug therapy, were considered as a comparative option for new approval [21,22]. This additional regulatory monitoring, with the call to report ADRs, can lead to an increased reporting rate and is known as the “Weber-effect”, described by JCP Weber in 1984, who postulated that the highest rate of spontaneous reports is observed two years after marketing authorization for the drug [23]. Hormonal contraceptives and their influence on possible psychiatric ADRs have been discussed in the media. A petition (regarding an extension of the package insert with regard to psychiatric symptoms) [24] led to scientific analyses [25] and a red-handed letter [26]. The topic was also of great interest outside Germany and led to increased ADR reporting rates [27,28]. Notably, media coverage caused the proportion of patient reports to rise from 23.5% to 98.6%. Patients reported significantly more ADRs per spontaneous report, with a median of 6, than healthcare professionals (median: 1). The reports were mainly related to anxiety and depression (38.8% vs. 10.6% before media coverage) and sexual dysfunction (47.3% vs. 6.9%) [28]. It is known that increased attention towards a drug class with a signal warning such as a red-handed-letter can lead to an increase in professionals and patients reporting ADRs. This so-called notoriety bias can skew results to overemphasize a special drug group [29].

Complementary to the spontaneous reports, systemic glucocorticoids and drugs with an influence on the central nervous system were disproportionately often suspected of causing an ADR in the ADRED study [14]. The ADRED study showed a high incidence of bleeding events (including gastrointestinal bleeding), which may have been caused by the use of antithrombotic agents, as well as systemic glucocorticoids in combination with or without NSAIDs [14,15]. Another study showed that topical glucocorticoids also have a high risk of ADRs, despite their exclusively local use; even short-term applications can alter the epidermis and can lead to skin atrophy. A critical risk–benefit assessment is necessary [30]. Many drugs with an influence on the central nervous system are considered as potentially inappropriate medications (PIMs) for geriatric patients [31,32,33] and therefore as a risk factor for ADRs [34,35,36]. Altered pharmacodynamics and pharmacokinetics with age [37] can lead to a higher potency due to a reduced volume of distribution and slower metabolization [38]. Dehydration can further aggravate this effect, which exhibited the greatest age dependence in the ADRED study [15]. In the future, it is crucial to scientifically examine these physiological correlations in the context of the changing global climate and to establish recommendations for action for patients and healthcare professionals.

In addition to PIMs, the increase in the number of drug therapies with increasing age due to comorbidities has been scientifically proven [39,40,41], and polypharmacy is a significant risk factor for the occurrence of ADRs [42]. In an ageing society, it is therefore more important than ever to alert healthcare professionals to the most common ADRs [43] and to work together with professional societies to find adequate solutions.

In addition to the age of patients and the associated increase in morbidity, other risk factors such as obesity must be considered. It is, for example, known that VEGF, as a growth factor for angiogenesis, is induced due to hypoxic conditions in visceral adipose tissue and can affect vascular reactions [44]. Further studies are needed to better assess the influence of metabolic status or, for example, nicotine abuse in order to detect risk modifiers for ADRs. The median number of suspected drugs for both methods is 1, thus showing that there is a strong tendency for one suspected drug to be identified [14]. However, there are differences in the number of drugs taken. In the ADRED study, all taken drugs were systematically collected and documented in the eCRF. In contrast to spontaneous reports, the documentation of concomitant drugs was mandatory and therefore can explain the significantly higher number of concomitant drugs [14]. In contrast, data on concomitant drugs in the spontaneous reports were often not even available. The federal standardized medication plan (BMP), Figure S3, which has been obligatory since October 2016 [45], is not used consistently [46] and is often neither up to date nor complete [45,46,47]. This makes it difficult for healthcare specialists to obtain a complete overview of the drugs taken and, consequently, to report them via the spontaneous report system [3]. If this issue is resolved, adding further mandatory fields to the digital questionnaires could significantly improve data quality.

The limitations of both methods must be considered. In the spontaneous reports, the high level of underreporting, the low data quality, and possible biases in reporting behaviour cannot be ignored. Prospective data collection can compensate for this but is not suitable for widespread use due to its high requirements and associated costs. In addition, possible exclusion criteria and the need for written informed consent can also lead to distortions.

## 5. Conclusions

Spontaneous reports remain an indispensable method for recording ADRs due to their ability to identify risk signals quickly and easily. However, their limitations make it necessary to continuously evaluate these data through scientific studies. Here, the high degree of conformity of the data allows quantitative questions to be answered reliably. However, the high costs involved prevent widespread expansion. In the future, it will be important to take advantage of the opportunities offered by advancing digitalization in healthcare. The introduction of digital patient records could significantly improve the exchange of information between all parties involved. However, there is also a need to focus more closely on data-driven evaluations of large healthcare datasets, e.g., from health insurance providers, to efficiently increase drug safety.

## Figures and Tables

**Table 1:** Characteristics of the comparison groups—spontaneous reports and the ADRED study.

	Spontaneous Reports *n* = 2022	Adult *n* = 1156 (57.2%)	Young-Old *n* = 647 (32.0%)	Old-Old *n* = 219 (10.8%)	ADRED *n* = 2215	Adult *n* = 731 (33.0%)	Young-Old *n* = 880 (39.7%)	Old-Old *n* = 604 (27.3%)
Age (years)	61 [48;73]	50 [38;57]	72 [68;76]	84 [81;87]	73 [58;80]	51 [38;58]	74 [70;77]	84 [82;87]
Sex, male	895 (44.3%)	476 (41.2%)	335 (51.8%)	84 (38.4%)	1115 (50.3%)	360 (49.2%)	495 (56.3%)	260 (43.0%)
Sex, not known	10 (0.5%)	6 (0.5%)	3 (0.5%)	1 (0.5%)	-	-	-	-
Number of suspected drugs	1 [1;1]	1 [1;1]	1 [1;1]	1 [1;1]	1 [1;2]	1 [1;2]	2 [1;2]	1 [1;2]
Number of drugs taken	2 [1;5]	1 [1;3]	3 [1;6]	4 [1;7]	7 [3;10]	3 [2;8]	8 [5;11]	8 [6;10]
Number of ADRs per report/case	2 [1;3]	2 [1;3]	2 [1;3]	2 [1;3]	2 [1;4]	2 [2;4]	2 [1;4]	2 [1;3]
Severity	Multiple choice possible ^2^							
Non-serious	950 (47.0%)	609 (52.7%)	297 (45.9%)	44 (20.1%)	261 (11.8%)	165 (22.6%)	58 (6.6%)	38 (6.3%)
Others ^1^	359 (17.8%)	226 (19.6%)	99 (15.3%)	34 (15.5%)	-	-	-	-
Disability	111 (5.5%)	55 (4.8%)	35 (5.4%)	21 (9.6%)	2 (0.1%)	0 (0.0%)	1 (0.1%)	1 (0.2%)
Hospitalization	562 (27.8%)	252 (21.8%)	199 (30.8%)	111 (50.7%)	1837 (82.9%)	525 (71.8%)	775 (88.1%)	537 (88.9%)
Life-threatening	223 (11.0%)	106 (9.2%)	73 (11.3%)	44 (20.1%)	107 (4.8%)	37 (5.1%)	45 (5.1%)	25 (4.1%)
Death	109 (5.4%)	29 (2.5%)	46 (7.1%)	34 (15.5%)	8 (0.4%)	4 (0.5%)	1 (0.1%)	3 (0.5%)

ADR: adverse drug reaction; divided into non-serious and serious (Others, Disability, Hospitalization, Life-threatening and Death) ADRs; ^1^ Others: other serious reactions; ^2^ within the spontaneous reports; continuous variables are shown as median [interquartile ranges, IQR], categorical variables are shown in absolute numbers (percentages).

**Table 2:** Drug groups with an OR (95% CI) > 1 for the spontaneous reports and the ADRED study; sorted by OR (95% CI) in descending order.

Spontaneous Reports (*n* = 2022) ∑_suspected_ = 2278; ∑_total_ = 5755; m = 29, z = 3.12	Suspected Drugs (*n*)	Proportion of All Suspected Drugs (%)	Total Drugs (*n*)	Proportion of All Total Drugs (%)	OR (95% CI)
Gynecological	56	2.5	60	1.0	21.88 (4.34–110.24)
Immunosuppressants	165	7.2	191	3.3	10.36 (5.34–20.13)
Antibiotics	168	7.4	202	3.5	8.06 (4.46–14.59)
Antineoplastic drugs	179	7.9	226	3.9	6.22 (3.71–10.45)
Lipid-modifying drugs	483	21.2	696	12.1	4.12 (3.14–5.42)
Sex hormones	36	1.6	57	1.0	2.64 (1.12–6.25)
Antithrombotics	296	13.0	486	8.4	2.58 (1.91–3.50)
ADRED study (*n* = 2215) ∑_suspected_ = 3985; ∑_total_ = 15,948; m = 28, z = 3.12					
Antineoplastic and immunomodulating drugs	591	14.8	692	4.3	20.45 (14.54–28.77)
Antithrombotics	763	19.1	1656	10.4	2.94 (2.49–3.47)
Antibiotics	118	3.0	254	1.6	2.65 (1.78–3.95)
Glucocorticoids (systemic)	87	2.2	196	1.2	2.43 (1.54–3.82)
Antipsychotics	77	1.9	176	1.1	2.36 (1.46–3.81)
Antiparkinsonian medications	46	1.2	112	0.7	2.11 (1.15–3.84)
Antidepressants	196	4.9	483	3.0	2.10 (1.57–2.83)
Opioids	129	3.2	349	2.2	1.79 (1.26–2.54)
Non-opioid analgesics	208	5.2	687	4.3	1.32 (1.01–1.72)

Drug groups found in more than 0.7% of all taken drugs in the dataset; ∑_suspected_: number of all suspected drugs: 2278 (Spontaneous Reports) and 3985 (ADRED study); ∑_total_: number of all taken drugs: 5755 (Spontaneous Reports) and 15,948 (ADRED study); odds ratio (OR) = ((n_suspected drug group_)/(∑_suspected_ − n_suspected drug group_))/((n_concomitant drug group_)/(∑_concomitant_ − n_concomitant drug group_)) with concomitant = total − suspected; 95% confidence interval (95% CI), Bonferroni-adjusted (m: number of tests) with m = 29 (Spontaneous Reports) and m = 28 (ADRED study) and a corresponding z value of 3.12 for both.

## Data Availability

Data are available upon request from the corresponding author.

## Supplementary Materials

The following supporting information can be downloaded at: https://www.mdpi.com/article/10.3390/jcm14175921/s1, Figure S1: The questionnaire to document spontaneous reports to the Drug Commission of the German Medical Association (AkdÄ); Figure S2: Recruiting flowchart in the ADRED study (Phase I); Figure S3: The federal standardized medication plan (BMP) in Germany.
