# Supplementary material for: Comparative Analysis of Drugs Frequently Suspected of Causing Adverse Drug Reactions Reported via the Spontaneous Reporting System Versus in a Prospective Multicentre Cohort Study in Hospital Emergency Departments
Source: J Clin Med. 2025 Aug 22;14(17):5921. doi: 10.3390/jcm14175921 (PMC12429642; doi:10.3390/jcm14175921)
Supplement: Supplementary file 1 [file jcm-14-05921-s001.zip › jcm-3783431-supplementary.docx]

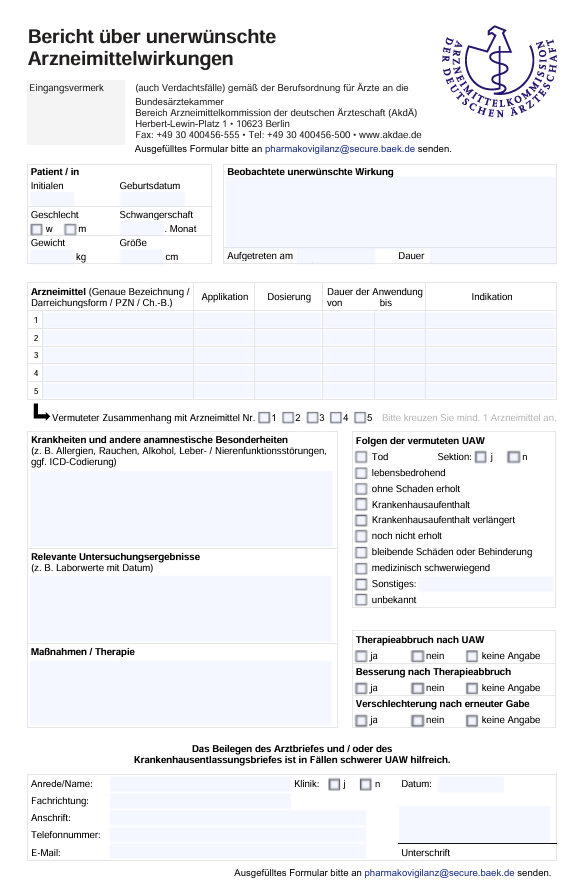


**Figure S1.** The questionnaire to document spontaneous reports to the Drug Commission of the German Medical Association (AkdÄ)


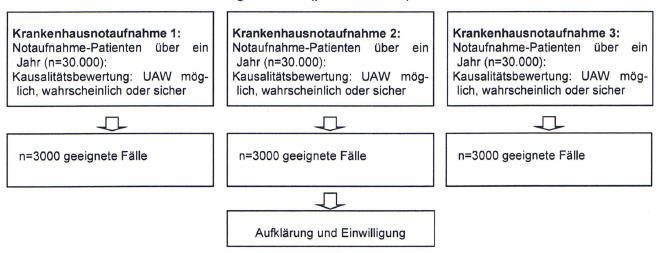


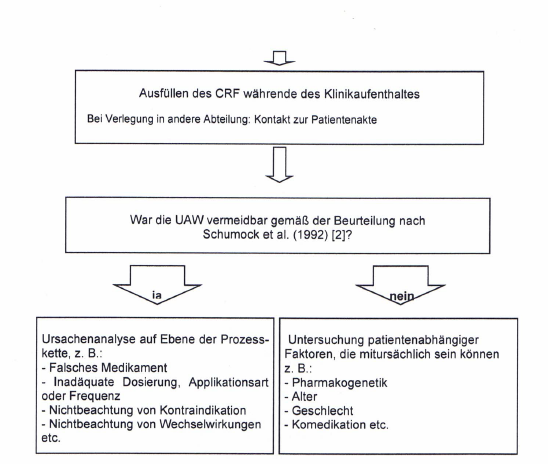


**Figure S2.** Recruiting flowchart in the ADRED study (Phase I).


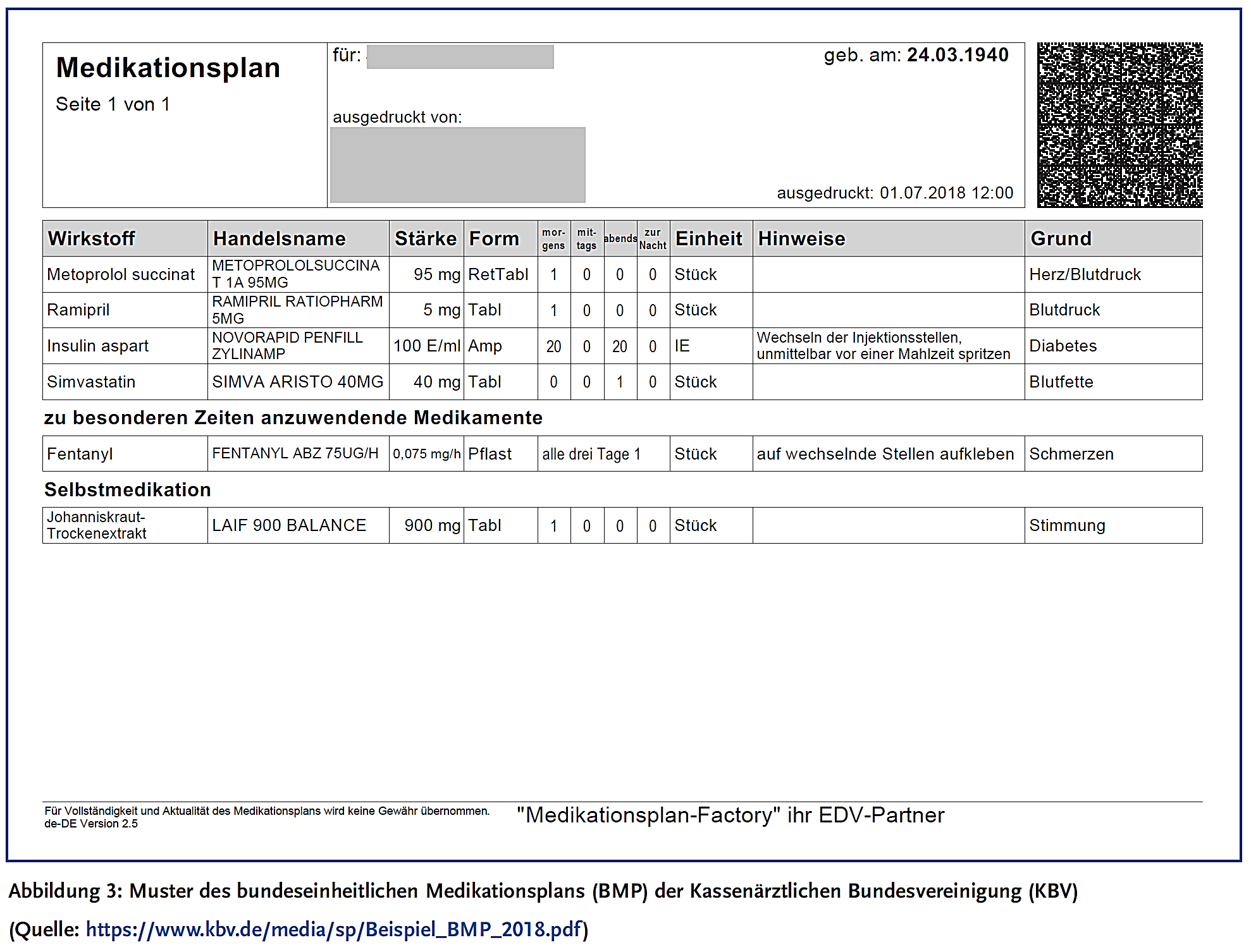


**Figure S3.** The federal standardized medication plan (BMP) in Germany.
